# Principles of Sustainable Healthy Diets in Worldwide Dietary Guidelines: Efforts So Far and Future Perspectives

**DOI:** 10.3390/nu13061827

**Published:** 2021-05-27

**Authors:** Daniela Martini, Massimiliano Tucci, James Bradfield, Antonio Di Giorgio, Mirko Marino, Cristian Del Bo’, Marisa Porrini, Patrizia Riso

**Affiliations:** 1 Department of Food, Environmental and Nutritional Sciences (DeFENS), Università degli Studi di Milano, 20133 Milan, Italy; massimiliano.tucci@unimi.it (M.T.); antonio.digiorgio1@studenti.unimi.it (A.D.G.); mirko.marino@unimi.it (M.M.); cristian.delbo@unimi.it (C.D.B.); patrizia.riso@unimi.it (P.R.); 2 NNEdPro Global Centre for Nutrition and Health, Cowley Road, Cambridge CB4 0WS, UK; j.bradfield@nnedpro.org.uk

**Keywords:** food-based dietary guidelines, healthy diets, sustainability, environmental impact

## Abstract

Food choices and eating behaviours have a large impact on both human and planetary health. Recently, the Food and Agricultural Organisation (FAO) of the United Nations and the World Health Organisation have developed a list of 16 guiding principles to achieve sustainable healthy diets (SHDs). They proposed that development of food-based dietary guidelines (FBDGs) should be a core element in the implementation of these SHDs in each country. The objective of this review is to explore the degree of alignment of current FBDGs to these guiding principles. A total of 43 FBDGs, written or translated into English, were collected from the online repository developed by the FAO and were analysed for their adherence to each of the guiding principles. Results were stratified for period of publication and geographical macro-area. Overall, there were high levels of inclusion of the factors related to health outcomes, especially in the most recent FBDGs. Conversely, environmental impact and socio-cultural aspects of diet were considered less frequently, especially in the older FBDGs. These results highlight the importance of revising FBDGs, especially to include emerging topics which represent the areas with the highest scope for improvement in the future versions of FBDGs. Replication of the present study in the coming years will be worthwhile to monitor improvements in the adherence of global FBDGs to the guiding principles of SHDs. The attainment of such a goal could promote a more rapid transition towards SHDs, as well as highlighting pivotal research trajectories to increase adoption and evaluate the impact on the food system.

## 1. Introduction

The Global Burden of Disease Study 2017 estimated that, in the adult population (older than 25 years), 22% of total deaths (11 million total) and 15% of disability-adjusted life-years (255 million total) are attributable to dietary risk factors [1]. While it is well-established that food choice and eating behaviours represent two important determinants of human health, they may also have an impact on the health of the planet at large [2,3]. Related is the increasing evidence indicating that food systems have a significant impact on the environment, given they are responsible for almost 30% of total greenhouse gas emission in developed countries [4,5]. Furthermore, around 40% of the Earth’s total land surface is currently used for agricultural practices, while it also accounts for approximately 70% of the total freshwater use [6,7]. As global populations continue to grow, it is estimated that the impact of diet on the environment will likely see an associated rise [6,8,9]. Thus, the measure of how healthy a diet is must be viewed through a double lens: on the one hand, a diet should focus on human health, through an appropriate intake and proportions of macronutrients to support energetic and physiological needs, as well as of micronutrients and hydration to meet the physiological needs of the body [10]. On the hand side, diet and the food system in general must take into account the planetary health as a major challenge [2,11,12].

Nutritionally inadequate diets represent a major driver of current climate change and will likely also exacerbate malnutrition, food insecurity and hunger, increasing the disease burden attributable to food availability and nutrition [12,13]. Undernutrition, obesity, climate change and fresh water depletion have been described as interconnected global challenges, with food systems an important underlying driver [14]. Thus, the definition and the implementation of healthy and sustainable diets are pivotal steps to tackle these critical issues. This has been recognised on a global level, with the concept of sustainability significantly represented in the United Nations (UN) Sustainable Development Goals (SDGs) [15]. The SDGs are a call for action by all countries, defined by the UN in 2015, and consist of 17 goals and 169 targets to promote prosperity while protecting the planet [16].

Recently, the Food and Agricultural Organisation (FAO) of the UN and the World Health Organisation (WHO) have developed a list of 16 guiding principles related to sustainable healthy diets (SHD), targeted at governments and other stakeholders in policy making and communication, to address the implementation of these issues [12]. These 16 principles are divided into three categories: health aspects, environmental impact and sociocultural aspects. In this document, the FAO and WHO also clearly state that one of the actions required to make SHD available, accessible, affordable, safe and desirable is the development of national food-based dietary guidelines (FBDGs) according to these principles.

FBDGs are an attempt to condense a large body of scientific evidence, translating the relations between dietary patterns and health into specific, culturally appropriate and actionable recommendations [17,18,19]. Since FBDGs are primarily intended for consumer information and education, they should be simple to understand and implement [20]. The development of national/regional FBDGs is promoted given the potential to improve human health alongside economically advantageous outcomes [21]. For FBDGs to be effective, they must be nationally or even regionally specific, making use of local data on consumption, dietary habits, customs and disease burden [22]. For this reason, each region or country must create tailor-made FBDGs to provide appropriate advice according to the needs of that particular population [23,24].

In 1992, during the International Conference on Nutrition, the FAO and the WHO encouraged governments to develop FBDGs as an important action to promote appropriate diets and healthy lifestyles [25]. Subsequently, in 1995, FAO and WHO elaborated and published the first general process for developing FBDGs [26], which still remains a point of reference. Later, in 2010, the European Food safety Authority (EFSA) provided further guidance on developing FBDGs for the diverse populations of Europe, following an approach consisting of seven steps [27]. 

In 2005, the WHO reported that 75 countries, 33 of which were in Europe, had developed FBDGs [28]. FBDGs must be periodically updated to account for advancements in nutritional research or changes in the needs of populations [27]. For example, Italy recently published the latest edition of its FBDGs (December 2018), dedicating an entire chapter to sustainability [29]. Thus, it is worth considering if the Italian, and other worldwide FBDGs, make reference to the principles of healthy and sustainable diets, as recently defined by the FAO and WHO.

Based on these observations, the aim of this review was to explore if the currently available FBDGs considered the 16 guiding principles for SHDs. This evaluation will allow (i) investigation of the current efforts made by different countries in fostering the adoption of healthy and sustainable diets and (ii) identification of possible gaps that should be considered in the next revisions of worldwide FBDGs.

## 2. Materials and Methods

### 2.1. Selection and Collection of Dietary Guidelines

In this study, we selected FBDGs of different countries using the online repository developed by FAO [30], where the majority of available world FBDGs can be reviewed. This repository was created in 2014, and since then, it has been continually updated to include every new revision (or first edition) of a country’s FBDG. In this repository, the countries are divided into geographical macro-areas (i.e., Africa, Asia and Pacific, Near East, Europe, Latin America and the Caribbean and North America).

The FBDG of each country listed by the FAO was collected from the repository; however, a further check for updates not listed was also completed. The search was performed in October 2020 and was updated in February 2021.

For this analysis, the only inclusion criterion was the availability of an English version of the FBDGs. An FBDG that was not in English but provided an English summary of the FBDG was also accepted. Conversely, exclusion criteria were: (i) FBDGs that constituted only of a one-page informative poster or brochure; (ii) FBDGs that were only available in a language other than English; (iii) FBDGs that only addressed the diets of children. For each country, only the most recent version of the FBDGs was considered. In cases where multiple versions existed for use by the general population and professionals/policy makers, only the former was considered. Finally, for each selected FBDG, only the guidelines addressing the generally healthy population were included in the final evaluation, except for guiding principle n° 1, which specifically refers to infants.

### 2.2. Data Collection from FBDG and Comparison with the Guiding Principles for Sustainable Healthy Diets

For each selected FBDG, the following information was collected: official name of the guideline, country, macro-area, year of the most recent revision and key messages. In case of reprint, the date of first publication of the FBDG was used for the analysis.

To assess the levels of adherence of each FBDG included in the analysis with the 16 guiding principles defined by FAO and WHO, each FBDG was searched for information or key messages matching with the relative guiding principles, listed in Table 1.

More specifically, key messages were defined for each guiding principle, considering the three main classes of guiding principles for SDHs proposed by the FAO and the WHO regarding: (i) health aspects; (ii) environmental impact; (iii) sociocultural aspects.

For each FBDG, the key messages were analysed for adherence with the 16 principles included in these three classes of principles of SHD. This analysis was performed by two independent reviewers (M.T. and A.D.G.). When needed, disagreement between the reviewers was solved through consultation with a third reviewer (D.M.) to reach a consensus.

Results were then summarised, stratifying by macro-areas and by year of publication. When describing the time of publication, three strata were used: (i) those published up to 2010; (ii) those published between 2011 and 2015; (iii) those published since 2016. The macro-areas were represented by Africa, Asia and Pacific, Near East, Europe, Latin America and the Caribbean and North America, as reported in the online repository by FAO [30].

However, due to the high variability in the number of countries for each macro-area, North America and Latin America were grouped together, as well as Asia and the Pacific and the Near East. Thus, macro-areas were finally grouped as: (i) Africa; (ii) Near East, Asia and the Pacific; (iii) Europe; (iv) North and Latin America.

## 3. Results

### 3.1. Dietary Guidelines: Geographical Distribution and Period of Publication

**Figure 1:**
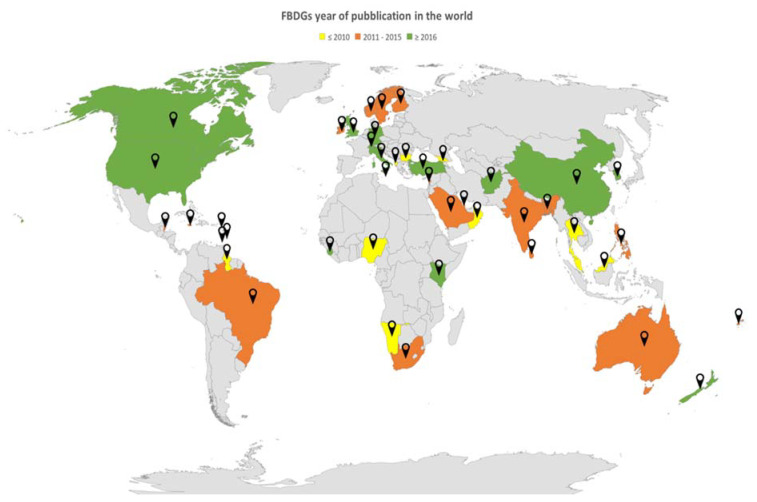
Geographical distribution and period of publication of the dietary guidelines. Legend: yellow: up to 2010; orange: 2011–2015; green: after 2016.

The number and the period of publication of the FBDGs included in the present evaluation are reported in Figure 1. The list of FBDGs with the related geographical macro-area and year or publication is reported in Supplementary Table S1.

A total of 43 FBDGs were available in English on the FAO online repository. In total, 14 FBDGs (33%) were developed in the most recent category (since 2016), while the majority were less recent: 18 (42%) were developed between 2011–2015 and 11 (26%) were developed before 2010. Regarding the geographical macro-area, most FBDGs were published by European countries (13 FBDGs), followed by the Near East, Asia and the Pacific (*n* = 16, including Australia and New Zealand), North and Latin America (*n* = 9) and Africa (*n* = 5).

### 3.2. Compliance of Total FBDGs to the Guiding Principles for Sustainable Healthy Diets

Guiding principles for SHDs and the compliance of the available FBDGs are presented in Table 1. The analysis revealed a high level of compliance from FBDGs with health-related guiding principles, but a critically low level of compliance with principles related to environmental impact and sociocultural aspects. More specifically, the guiding principles related to health aspects (i.e., principles 1–8) were included from a minimum of 56% of the time for principle n° 1 and up to a maximum of 95% and 98% for principles n° 2 and 3, respectively. Conversely, aspects related to the environmental impact (i.e., principles 9–13) were included at far lower rates, ranging from 5% (principle n° 11) to 23% for principle n° 13. Finally, we found high heterogeneity in the levels of compliance with guiding principles related to socio-cultural aspects (i.e., principles 14–16). Indeed, compliance ranged from a minimum of 7% for principle n° 16 to a medium-high level of compliance (53%) observed for principle n° 14.

### 3.3. Compliance of FBDGs with Guiding Principles for Sustainable Healthy Diets, by Publication Time and Geographical Macro-Area

#### 3.3.1. Health Aspects

**Figure 2:**
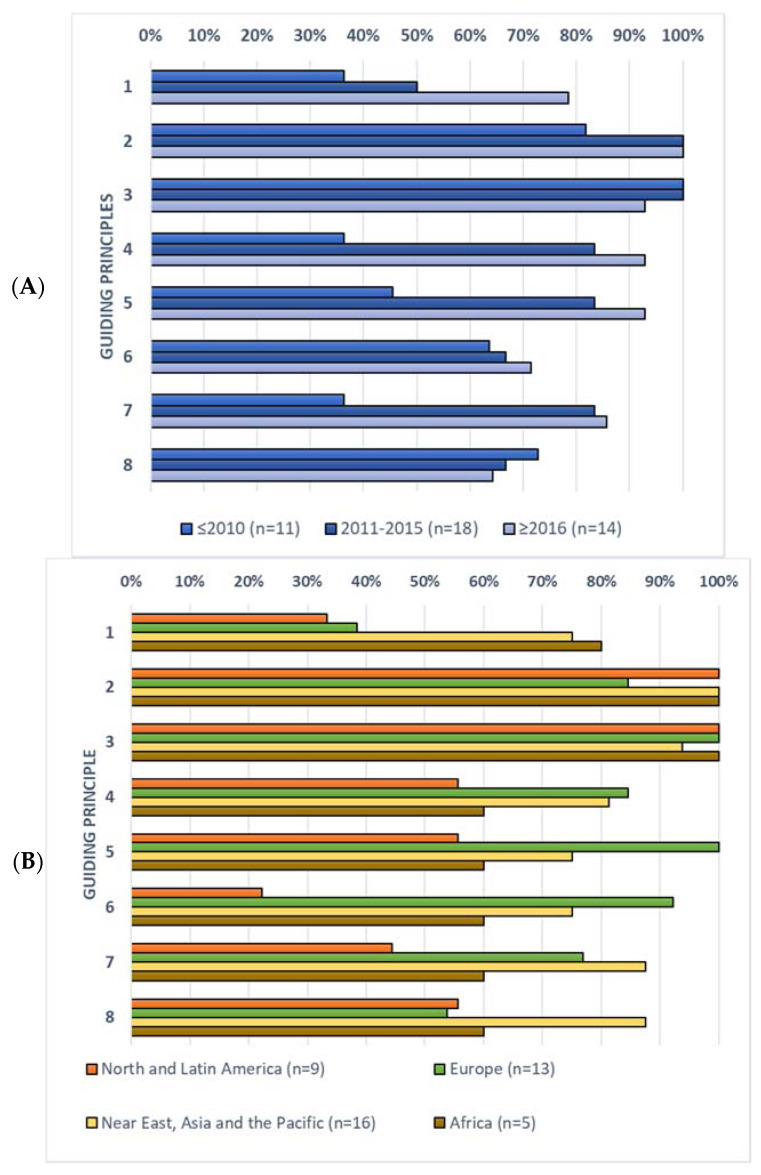
Rate of inclusion of the guiding principles for sustainable healthy diets related to health aspects by the 43 FBDGs included in the evaluation, divided by period of publication (**A**) and geographical macro-area (**B**).

The inclusion of the guiding principles for SHD related to the health aspect (i.e., principles 1–8), by period of publication and geographical macro-area, is summarised in Figure 2.

Regarding differences based on time of publication, these guiding principles were more widely addressed in the more recent FBDGs (Figure 2A). All principles were most often considered in the FBDGs produced between 2011–2015 and those produced since 2016 compared to those published before 2010. For instance, principle n° 2 was included in 100% of FBDGs published between 2011 and 2015 and since 2016, and in 82% in the period before 2010. The only exception was for guiding principle n° 8, related to food safety and hygiene (i.e., SDHs contain minimal levels, or none if possible, of pathogens, toxins and other agents that can cause foodborne disease), which was most often considered in the FBDGs published before 2010 (73%) compared to those of the other two time periods (67%).

Regarding geographical macro-areas (Figure 2B), a high variability was observed among the rate of inclusion of the guiding principles in the four different macro-areas. The lowest rate of inclusion was in the American FBDGs (on average 58% of inclusion) and the highest in the ones from the Near East, Asia and the Pacific area (84%). Intriguingly, principle n° 1, related to breastfeeding, was considered in 75% and 80% of the FBDGs published in African and the Near East, Asia and the Pacific, respectively, but only in the 38% and 33% of the European and American versions, respectively. Conversely, only two American FBDGs (22%) considered principle n° 6, which was included in 92% and 75% of the European Asiatic FBDGs and in those from the Near East, Asia and the Pacific, respectively.

#### 3.3.2. Environmental Impact

**Figure 3:**
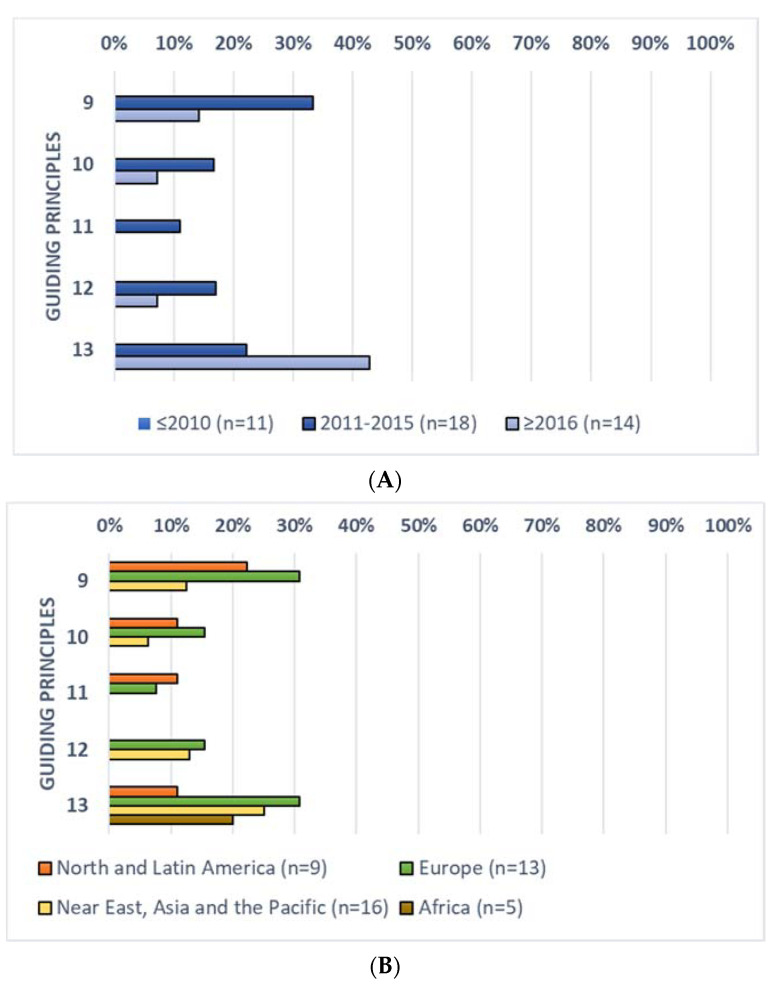
Rate of inclusion of the guiding principles for sustainable healthy diets related to environmental impact by the 43 FBDGs included in the evaluation, divided by period of publication (**A**) and geographical macro-area (**B**).

The inclusion of the guiding principles for SHD related to the environmental impact (i.e., principles 9–13) by period of publication and geographical macro-area is summarised in Figure 3.

In short, similarly to health aspects, these guiding principles were more widely considered in the more recent FBDGs (i.e., between 2011 and 2015 and since 2016) compared to the older versions (Figure 3A). In fact, none of the FBDGs published before 2010 included in this analysis considered these five aspects (i.e., principles 9–13), while they were considered on average by 20% and 14% of the FBDGs published between 2011–2015 and since 2016, respectively. The principles included in the lowest number of FBDGs were principles n° 11 (included in 11% and 0% of FBDGs published between 2011–2015 and since 2016, respectively) and n° 10 and 12 (17% and 7% in the two time periods, respectively). Conversely, among principles related to environmental impact, n° 13 was considered in the highest number of FBDGs (22% and 43% in the two time periods, respectively) followed by principle n° 9 (33% and 14% respectively).

Dividing results by geographical macro-areas (Figure 3B), African FBDGs showed the lowest rate of inclusion, with only one country including one of the five guiding principles. Conversely, the highest rate of inclusion was observed in the European ones, on average considered in 20% of the FBDGs.

#### 3.3.3. Socio-Cultural Aspects

**Figure 4:**
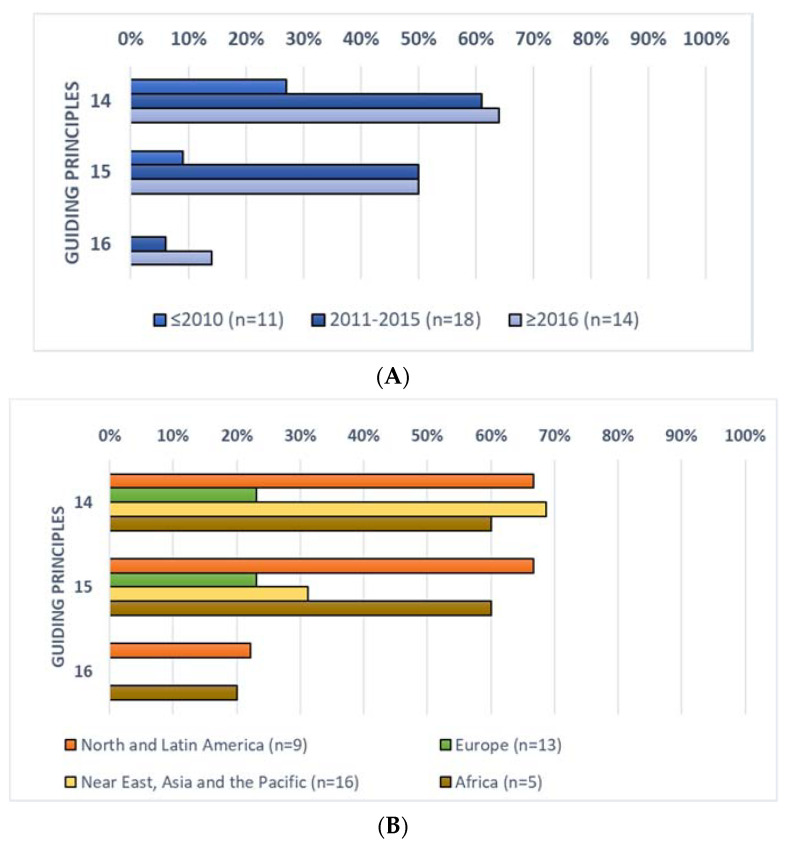
Rate of inclusion of the guiding principles for SHD related to socio-cultural aspects by the 43 FBDGs included in the evaluation, divided by period of publication (**A**) and geographical macro-area (**B**).

The inclusion of the guiding principles for SHD on socio-cultural aspects by period of publication and geographical macro-area is summarised in Figure 4.

In general, socio-cultural aspects (principles 14–16) were considered only in a few FBDGs, in a manner similar to what was observed for environmental impacts. Percentages of FBDGs, including these principles, ranged from 7% for principle n° 16 to 53% for n° 14. Similar to environmental aspects, these principles were mostly considered in FBDGs published between 2011–2015 and since 2016 compared to those published before 2010 (Figure 4A). For instance, principle n° 14 was considered in only 27% of FBDGs published before 2010, but in the 61% and 64% of those published between 2011–2015 and since 2016, respectively.

In relation to geographical distribution, the lowest rate of inclusion was observed in the European countries, where on average the guiding principles were considered in 15% of the FBDGs (ranging from 0% of the principle n° 16 to 23% for principle n° 14), while American and African FBDGs showed the highest rate (52% and 47%, respectively) (Figure 4B).

## 4. Discussion

In the present review, we aimed to provide an overview of worldwide FBDGs, investigating the extent to which FBDGs address the guiding principles for SHDs proposed by the FAO and WHO. The analysis of key messages within the 43 FBDGs included in the present evaluation highlighted that, as expected, the principles related to the health aspects of the diet were considered by a large majority of FBDGs. As already observed, this is likely because disease prevention and nutrient recommendations were considered the most pressing issues for the establishment of FBDGs in the past decades [31].

Interestingly, there was greater adherence with all principles in the most recent FBDGs, highlighting the importance of revising these documents, taking into consideration new scientific evidence, social developments and emerging topics that may affect dietary behaviour.

Despite overall quite high levels of compliance of FBGDs with health aspects, guiding principle n° 1, related to breastfeeding and complementary feeding, was included in only 56% of total FBDGs. These were mostly considered in the most recent FBDGs and in those by African countries and countries of the Near East, Asia and the Pacific macro-area. This point could represent a possible gap in the current FBDGs that should be included in the future revisions. Poor knowledge about breastfeeding factors contributes to the low rates of exclusive breastfeeding in many countries around the world and FBDGs may represent a viable tool to facilitate a change in practice. In turn, this may help to achieve the goal of having at least 50% of children exclusively breastfed for the first six months of life [32].

Intriguingly, the principle related to adequate energy intake was represented differently in the various macro-areas. Developed countries focused more on highlighting the importance of not exceeding energy intake and maintaining a healthy body weight, while developing countries tended to focus more on the prevention of undernutrition or nutritional deficiencies. However, it is noteworthy that despite the prevalence of undernutrition and the scale of the problem in developing countries, the prevalence of obesity is increasing and, in some countries, it has reached levels similar to that of developed countries [33]. Thus, the co-existing double burden of over- and undernutrition should receive more attention in the FBDGs, especially for the countries affected.

Despite differences among countries, our analysis indicates that most FBDGs recommend adherence to plant-based dietary patterns, high in wholegrains, fruit and vegetables, nuts and legumes and with a low to moderate amounts of animal products such as meat. As already observed by Herforth and colleagues [17], the consumption of a variety of foods, with differing proportions of the various food groups, and the reduction of some specific nutrients (e.g., sugar and salt) appears nearly universal across guidelines, despite not all countries stating this explicitly. Similarly, many FBDGs recognise the role of healthy diets in the prevention of chronic diseases. Overall, this advice is largely comparable to the Mediterranean dietary pattern [34], which has been reported to have the same beneficial effects on several risk factors of diseases [35,36] and a lower environmental impact compared to the average current diet of the Italian population [37,38].

In comparison to health aspects, few FBDGs included instructions around environmental impacts of diet, with the lowest rates in Africa and in FBDGs published before 2010, once again highlighting the need to revise these documents to take into consideration new scientific evidence. These results are in line with those recently observed by Bechthold and co-workers, who have found that sustainability was considered only in the Swedish FBDGs, while the other 33 dietary guidelines analysed did not discuss the topic [31]. However, it is noteworthy that some revised versions of FBDGs have been developed in more recent years. For instance, FAO highlighted that the FBDGs of other three countries (Germany, Brazil and Qatar), in addition to Sweden, explicitly reference or take account of environmental factors in their main messaging [27], despite to a different extent. Qatar dedicated point 7 of their FBDG to the topic “Eat Healthy while Protecting the Environment,” mostly focusing on the reduction of food waste and on the consumption of foods produced locally and regionally. Regarding Brazil, the FBDG mostly highlighted that healthy diets derive from socially and environmentally sustainable food systems and that dietary recommendations should consider the impact of the means of production and distribution of food on social justice and environmental integrity. Recent guidelines that considered the environmental aspects of the diets also include the last revision of the Italian FBDG that dedicated an entire chapter to aspects related to sustainability (e.g., reduction of food waste) [29].

Overall, the poor alignment with environmental improvements may be related to the fact that some environmental principles are more easily implementable at a population level (e.g., the reduced use of plastic), compared to others (e.g., minimal use of antibiotics in food production) that require policy change.

Among the various environmental factors, guiding principle n° 13, related to food waste and losses, was included most frequently (23% of total FBDGs). It is estimated that food waste and losses account for about 15% of the total environmental impact of food production in Europe [39], representing an associated economic loss for all actors throughout food supply chains. The majority of food waste occurs during production (73%) [39], but since food waste also significantly occurs at individual and household levels, further efforts should be made to align FBDGs with this principle. Furthermore, reducing food waste is an additional target of the SDGs, which have been agreed on by countries around the world [15].

We also found that overall, FBDGs, especially the less recent ones, do not align well with the sociocultural aspects of food and diet, despite occurring more frequently than aspects related to the environmental impact. However, a high variability was observed among principles, with the highest compliance observed for principle n° 14, related to respect of the local culture, culinary practices, knowledge and consumption patterns. In this context, the Mediterranean diet has been shown to be a clear example of a sustainable diet, so that it was recognised as an Intangible Cultural Heritage of Humanity by the United Nations Educational, Scientific and Cultural Organisation (UNESCO), which described the Mediterranean diet as “a ‘way of life–lifestyle’, a set of skills, knowledge, rituals, symbols and traditions, ranging from the landscape to the table. Eating together is the foundation of the cultural identity and continuity of communities throughout the Mediterranean basin. The Mediterranean diet emphasises values of hospitality, neighbourliness, intercultural dialogue and creativity, and a way of life guided by respect for diversity” [40].

Several socio-cultural aspects can affect food choices and dietary behaviours. For instance, these include accessibility to shops and food sources, depending on resources such as transport and geographical location [41]. Such factors influence food availability and their affordability. Indeed, it is reported that low-income groups tend to consume poorer quality diets, low in fruits and vegetables [42]. However, it has also been reported that a healthy and environmentally sustainable diet can be affordable for groups of various economic prosperity, while these issues were strictly related to cultural and knowledge barriers [43,44]. In this regard, guiding principle n° 15, related to accessibility and desirability, has been included more widely in Africa, America and the Near East, Asia and the Pacific rather than in Europe. This is likely because there were more FBGDs from middle/low-income countries in this macro-area rather than in Europe which emphasized the affordability recommendation (e.g., “cultivate your own vegetable garden”). Other important determinants of food choices are related to the social setting and context [45]. Gender has been described as an important factor, depending on the general culture scenario, for instance with gender being the strongest determinant of time spent cooking [46]. Gender equality is considered essential for a world free from hunger, malnutrition and poverty [47]; thus, the low level of alignment of FBDGs for this principle (addressed in only 7% of total FBDGs) shows that avoiding adverse gender-related impacts, especially with regard to time allocation, should hopefully be considered in the next revisions of the FBDGs.

Overall, the FBDGs included in this analysis do not align well with the principles laid out by the FAO and WHO. This is true for all categories, especially those factors related to the environmental impact of diet, and these factors represent the main areas that countries should seek to improve in subsequent revisions of the FBDGs, especially considering that current FBDGs are not in line with a set of global environmental targets related to climate change and environmental resource use [48].

Our study has some limitations worth noting. First, we considered only FBDGs developed or translated in the English language; thus, we cannot exclude the possibility that FBDGs available in other languages could have included the guiding principles to a greater degree. Secondly, we evaluated the presence of key messages within FBDGs to assess the alignment with the associated guiding principles, not considering the level of detail provided in the dietary recommendations.

However, our study also has several strengths. First of all, in contrast to other studies, we considered FBDGs from all over the world and not only from one continent or geographical area [19,49,50]. Additionally, we focused on several categories of factors related to health and sustainable diets, to provide a clear overview of the efforts made so far by the various countries included in the analysis. Furthermore, we stratified FBDGs to consider both time of publication and geographical macro-area. This approach allowed us to perform a comprehensive comparison of alignment of the FBDGs and to better elucidate which factors are the main drivers influencing the rate of inclusion of the different principles for SHDs.

## 5. Conclusions

In conclusion, the present review highlights that current FBDGs are poorly aligned with the guiding principles for SDHs, as proposed by FAO and WHO. This is especially true for factors related to socio-cultural aspects and environmental impacts of the diets, while health aspects showed higher rates of alignment. In the coming years, it would be useful to replicate this approach to monitor improvements in the alignment of FBDGs. It would also be worth investigating the effectiveness of these FBDGs in improving knowledge among the general population on this topic, as well as in improving dietary behaviours. In this regard, the availability of up-to-date FBDGs could help promoting a more rapid transition towards a more sustainable food system as well as supporting the development of multidisciplinary research, enabling both the improvement—in terms of environmental and nutritional sustainability—of the food chain from production to consumption, and investigating how to increase the adoption of healthy diets and evaluate their impact.

## Figures and Tables

**Table 1:** Guiding principles for healthy and sustainable diets [12].

Category	Number	Guiding Principle on Sustainable Healthy Diets	N° of FDBGs Including the Principle	% (*n =* 43)
Health aspects	(1)	*“… start early in life with early initiation of breastfeeding, exclusive breastfeeding until six months of age and continued breastfeeding until two years and beyond, combined with appropriate complementary feeding.”*	24	56%
	(2)	*“… are based on a variety of unprocessed or minimally processed foods, balanced across food groups, while restricting highly processed food and drink products.”*	41	95%
	(3)	*“… include wholegrain, legumes, nuts and an abundance and variety of fruits and vegetables.”*	42	98%
	(4)	*“… can include moderate amounts of eggs, dairy, poultry and fish and small amounts of red meat.”*	32	74%
	(5)	*“… include safe and clean drinking water as the fluid of choice.”*	33	77%
	(6)	*“… are adequate (i.e., reaching but not exceeding needs) in energy and nutrients for growth and development and to meet the needs for an active and healthy life across the lifecycle.”*	29	67%
	(7)	*“… are consistent with WHO guidelines to reduce the risk of diet-related NCDs, and ensure health and wellbeing for the general population.”*	31	72%
	(8)	*“… contain minimal levels, or none if possible, of pathogens, toxins and other agents that can cause foodborne disease.”*	29	67%
Environmental impact	(9)	*“… maintain greenhouse gas emissions, water and land use, nitrogen and phosphorus application and chemical pollution within set targets.”*	8	19%
	(10)	*“… preserve biodiversity, including that of crops, livestock, forest-derived foods and aquatic genetic resources, and avoid overfishing and overhunting.”*	4	9%
	(11)	*“… minimise the use of antibiotics and hormones in food production.”*	2	5%
	(12)	*“… minimise the use of plastics and derivatives in food packaging.”*	4	9%
	(13)	*“… reduce food loss and waste.”*	10	23%
Sociocultural aspects	(14)	*“… are built on and respect local culture, culinary practices, knowledge and consumption patterns, and values the way food is sourced, produced and consumed.”*	23	53%
	(15)	*“… are accessible and desirable.”*	17	40%
	(16)	*“… avoid adverse gender-related impacts, especially with regard to time allocation (e.g., for buying and preparing food, water and fuel acquisition).”*	3	7%

## Supplementary Materials

The following are available online at https://www.mdpi.com/article/10.3390/nu13061827/s1, Table S1: Countries of food-based dietary guidelines (*n* = 43) included in the present analysis, with related geographical macro-area, year of publication and specific title [30].
