# Supplementary material for: Principles of Sustainable Healthy Diets in Worldwide Dietary Guidelines: Efforts So Far and Future Perspectives
Source: Nutrients. 2021 May 27;13(6):1827. doi: 10.3390/nu13061827 (PMC8228140; doi:10.3390/nu13061827)
Supplement: Supplementary file 1 [file nutrients-13-01827-s001.zip › nutrients-1186745-supplementary.pdf]

nutrients

MDPI

Geographical macro-area	Country	Year	Title of the guidelines
Africa	Kenya	2017	National guidelines for healthy diets and physical activity
	Namibia	2000	Food & nutrition guidelines for Namibia – Food choice for a healthy life
	Nigeria	2001	Food based dietary guidelines for Nigeria – A guide to healthy eating
	Sierra Leone	2016	Sierra Leone food based dietary guidelines for healthy eating
	South Africa	2013	Food-based dietary guidelines for South Africa
Europe	Albania	2008	Recommendation on healthy nutrition in Albania
	Bulgaria	2006	Food based dietary guidelines for adults in Bulgaria
	Finland	2014	EAT GOOD - The Finnish dietary guidelines
	Georgia	2005	Healthy eating – The main key to health
	Germany	2017	10 guidelines of the German Nutrition Society (DGE) for a wholesome diet
	Ireland	2011	Scientific Recommendations for healthy eating guidelines in Ireland
	Italy	2019	Linee guida per una sana alimentazione
	Malta	2015	Healthy eating – The Mediterranean way!
	Norway	2014	The Norwegian dietary guidelines
	Sweden	2015	Find *your* way to eat greener, not too much and be active
	Switzerland	2016	Swiss food pyramid - Recommendations for a healthy and enjoyable adult diet
	Turkey	2016	Turkey dietary guidelines
	United Kingdom	2016	The Eatwell guide – Helping you eat a healthy, balanced diet
Near East, Asia and the Pacific	Afghanistan	2016	National food-based dietary guidelines for Afghans
	Australia	2013	Australian dietary guidelines
	Bangladesh	2013	Dietary guidelines for Bangladesh

	China	2016	Chinese dietary guidelines
	Fiji	2013	Food & health guidelines for Fiji
	India	2011	Dietary guidelines for Indians – A manual
	Lebanon	2013	Lebanon food-based dietary guidelines
	Malaysia	2010	Malaysian dietary guidelines
	New Zealand	2020	Eating and activity guidelines for New Zealand adults
	Oman	2009	The Omani guide to healthy eating
	Philippines	2012	Nutritional guidelines for Filipinos
	Qatar	2015	Qatar dietary guidelines
	Republic of Korea	2016	9 general dietary guidelines for Koreans
	Saudi Arabia	2012	Dietary guidelines for Saudis – The healthy food palm
	Sri Lanka	2011	Food based dietary guidelines for Sri Lankas
	Thailand	2007	Food based dietary guidelines for Thai
North and Latin America	Barbados	2017	Food based dietary guidelines for Barbados – Revised edition (2017)
	Belize	2012	Food-based dietary guidelines for Belize
	Brazil	2015	Dietary guidelines for the Brazil population
	Canada	2019	Canada's dietary guidelines
	Grenada	2006	Food Based dietary guidelines for Grenada
	Guyana	2004	Food based dietary guidelines for Guyana
	Jamaica	2015	Food based dietary guidelines for Jamaica 2015 – Healthy eating active living
	Saint Kitts and Nevis	2010	Food based dietary guidelines St. Kitts & Nevis
	United States	2020	Dietary guidelines for Americans 2020 - 2025

Suppl. Tab.1 Countries of food-based dietary guidelines (n=43) included in the present analysis, with related geographical macro-area, year of publication and specific title [30].
